# Student and Graduate Perceptions of Their Development of the Core Attitudes From the British Pharmacological Society's Undergraduate Pharmacology Curriculum

**DOI:** 10.1002/prp2.70291

**Published:** 2026-06-18

**Authors:** Sophie G. Reed, Aidan Seeley, Nicola Williams, John Broad

**Affiliations:** ^1^ Swansea University UK; ^2^ The British Pharmacological Society UK; ^3^ King's College London UK

**Keywords:** curriculum, education, pharmacology, undergraduate

## Abstract

The British Pharmacological Society's undergraduate core curriculum defines 18 core attitudes that pharmacology students should have after the completion of their degree. While the attainment of pharmacological knowledge and application of skills have previously been evaluated, assessment of core attitudes has not previously been reported. This study investigated student and graduate perceptions of their development of the core attitudes following the introduction of the core curriculum. An anonymous online survey was distributed via members of the British Pharmacological Society's Educator Network to current undergraduate pharmacology students and graduates, who were asked to rate their agreement, using a five‐point Likert scale, with statements indicating whether their degree contributed to the development of each of the 18 core attitudes. A total of 118 students and 70 graduates participated. Both groups reported predominantly positive perceptions across all attitudes, ranging from 54.2%–93.2% (64/118–110/118) in students and 57.1%–91.4% (40/70–64/70) in graduates, with the strongest agreement observed for appreciation of the societal relevance of pharmacology. Although overall response distributions differed significantly between students and graduates, differences were limited to a small number of statements, with graduates showing greater variability and more polarized responses. The highest proportion of negative responses in both groups related to identifying employment opportunities and pursuing career goals. Collectively, these findings indicate that pharmacology programmes are largely effective in fostering core attitudes, while highlighting opportunities for educators to reflect on and further strengthen career development and professional preparation within undergraduate curricula.

## 1 Introduction

The British Pharmacological Society's undergraduate pharmacology core curriculum, launched in 2016, is divided into three sections: core knowledge, core skills, and core attitudes [1]. The core curriculum outlines what graduates should know, be able to do, and how they should think and behave upon completion of an undergraduate degree in pharmacology: (i) the 32 core knowledge statements provide the disciplinary foundation required to understand, interpret, and communicate pharmacological principles; (ii) the 21 core skills statements emphasize the application of this knowledge through experimental, analytical, and communication competencies; and (iii) the 18 core attitude statements represent behaviors and professional values that pharmacology graduates are expected to develop during the degree, recognizing that graduate success depends not only on knowledge and skills, but also on professional values and behaviors [2]. In 2022, the Society developed broad learning outcomes for each core knowledge statement, along with indicative assessment examples for evaluating core skills [3]. Alongside this, to support the development and evaluation of core attitudes, the Society created a reflective proforma for students and identified evidentiary activities that would demonstrate these attitudes [3].

While studies have evaluated the attainment of pharmacological knowledge and/or skills [4, 5], evaluation of core attitudes in pharmacology students and graduates has not yet been evaluated. Understanding how effectively these core attitudes are cultivated during a pharmacology degree, and whether perceptions differ between current students and graduates, can provide insight into the educational impact of pharmacology programmes and highlight potential areas for curricular improvement.

This study aimed to evaluate student and graduate perceptions on their development of these core attitudes after the introduction of the pharmacology core curriculum [1].

## 2 Methods

### 2.1 Participants and Survey Design

Members of the British Pharmacological Society's Educator Network were contacted by email and invited to complete a short Microsoft Forms questionnaire to indicate whether they would be willing to distribute a survey to current students and/or graduates. In total, 34 educators confirmed their ability to disseminate the survey, representing 16 UK higher education institutions, eight institutions outside the UK, and three unspecified institutions.

Through these educators, the anonymous survey was disseminated to students and graduates using Microsoft Forms between May and December 2025. Eligibility was restricted to individuals aged 18 years or older who were either currently enrolled in an undergraduate pharmacology degree or graduates who had completed an undergraduate pharmacology degree in 2017 or any subsequent year, thereby excluding graduates who completed their studies prior to the 2016 launch of the undergraduate pharmacology core curriculum.

Respondents were asked to indicate whether they were a student or graduate and whether they were based within or outside of the United Kingdom. No other demographic data were collected to ensure respondent anonymity and reduce the risk that responses could be linked to specific institutions or individuals within a relatively small disciplinary community. Respondents were then asked their level of agreement with the 18 core attitude statements from the core curriculum using a five‐point Likert scale (strongly agree, agree, neither agree nor disagree, disagree, strongly disagree). Statements were prefaced with either “my degree is developing my…” for current students or “as a result of my degree, I developed…” for graduates. The survey collected no identifiable information, and responses were fully anonymous. Participants were informed that completion of the survey implied consent to participate and that due to the anonymous nature of data collection, withdrawal would not be possible once the questionnaire had been submitted.

All questions within the survey were required to be completed for respondents to submit their responses and, in total, 118 current students (90 UK‐based and 28 not UK‐based) and 70 graduates (67 UK‐based and three non‐UK‐based) completed the survey.

Ethical approval for this study was granted by Swansea University Medical School Research Ethics Committee (Reference: 2 2024 11511 11323).

### 2.2 Data Analysis

Frequencies and percentages for the core attitude statements were calculated for each Likert scale response category. Descriptive statistics were used to summarize responses, whereby responses were grouped into three categorical outcomes: positive (strongly agree/agree), neutral (neither agree nor disagree), and negative (disagree/strongly disagree), with data presented as percentages.

Comparisons between current students and graduates were performed using Fisher's exact test to assess differences in the distribution of responses between groups. Statistical analyses were conducted in GraphPad Prism 10 with statistical significance set at *p* < 0.05.

## 3 Results

**FIGURE 1:**
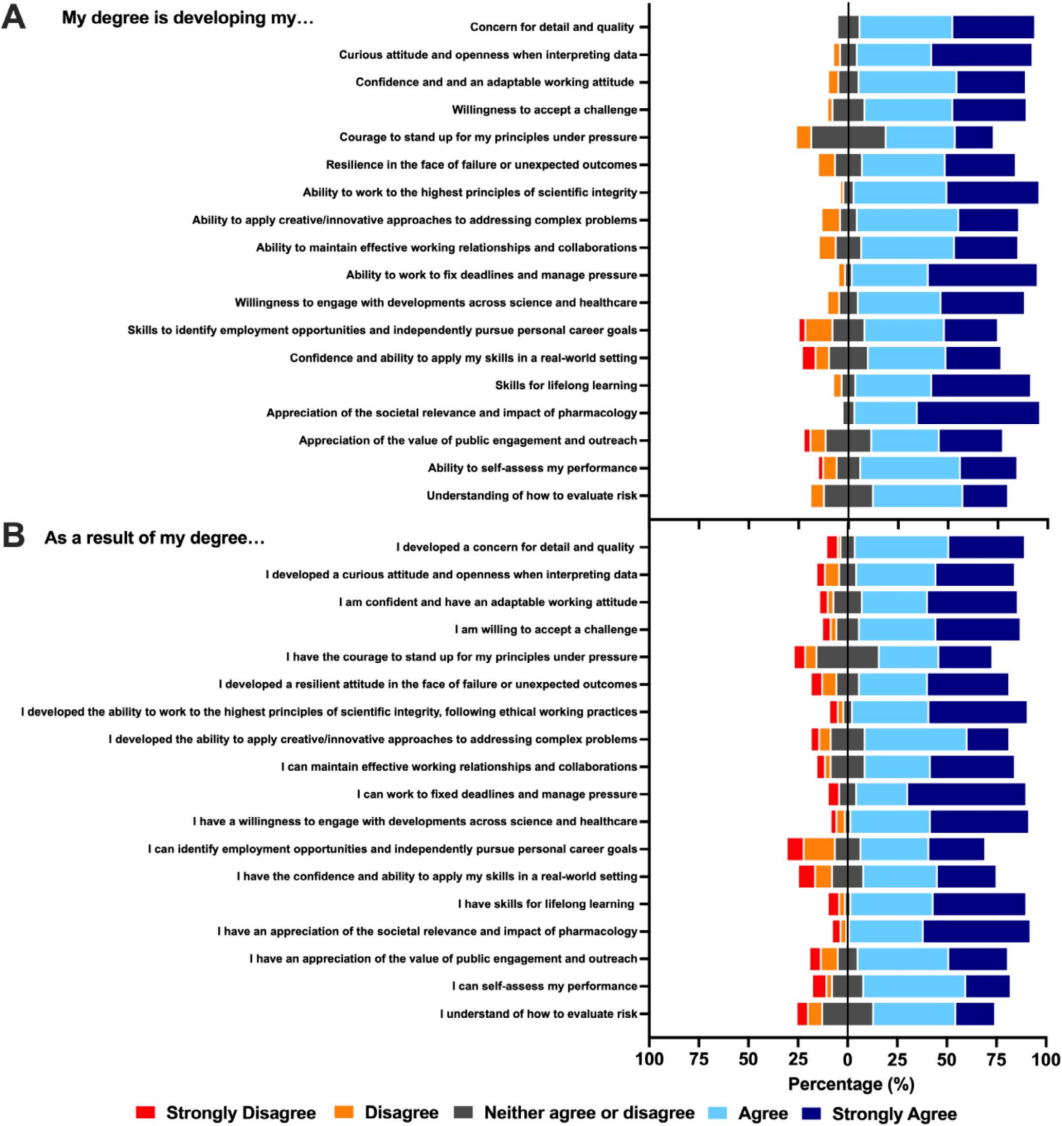
Self‐perceptions of students and graduates on their development of the core attitude statements from the British Pharmacological Society's core undergraduate pharmacology curriculum. (A) Student and (B) graduate responses were recorded on a five‐point Likert scale for each of the core attitude statements. Data are presented as percentages of total responses for each attitude, with positive responses (strongly agree and agree) shown to the right of the central axis and negative responses (disagree and strongly disagree) to the left, based on 118 student responses and 70 graduate responses.

Across all attitude statements, positive responses ranged from 54.2%–93.2% (64/118–110/118) in students and 57.1%–91.4% (40/70–64/70) in graduates. Neutral responses ranged from 3.4%–37.3% (4/118–44/118) in students and 1.4%–31.4% (1/70–22/70) in graduates, while negative responses were low in both groups, ranging from 0.8%–16.9% (1/118–20/118) in students and 5.7%–24.3% (4/70–17/70) in graduates (Figure 1).

The most positively perceived core attitude statement, in which 93.2% (110/118) of students and 91.4% (64/70) of graduates agreed or strongly agreed, was “…appreciation of the societal relevance and impact of pharmacology” (Figure 1). Similarly, 93.2% (110/118) of students also agreed or strongly agreed that their degree was developing an “…ability to work to the highest principles of scientific integrity, following ethical working practices” and an “…ability to work to fixed deadlines and manage pressure” (Figure 1A).

Comparison of the overall distribution of responses between students and graduates revealed a significant difference between groups (Fisher's exact test, *p* < 0.0001). Analysis of individual statements identified significant differences in responses between students and graduates to three of the core attitude statements; although both groups tended to respond positively, graduates consistently showed a more variable and polarized pattern of responses. For “…maintain effective working relationships and collaborations,” graduates showed a small cluster of “Strongly Disagree” responses (*p* = 0.025), which was absent in students. A similar pattern was observed for “…ability to work to fixed deadlines and manage pressure” (*p* = 0.007) and “…willing to accept a challenge” (*p* = 0.049), with graduates having more negative responses compared to current students.

For both students and graduates, the core attitude statement with the highest proportion of negative responses was “…identify employment opportunities and independently pursue personal career goals” (16.9% [20/118] of students and 24.2% [17/70] of graduates). In both groups, “…courage to stand up for my principles under pressure” was the statement with the most neutral responses (37.3% [44/118] of students and 31.4% [22/70] of graduates), followed by “…how to evaluate risk” (24.6% [29/118] of students and 25.7% [18/70] of graduates).

Collectively, these findings suggest that graduates hold slightly more diverse perspectives, occasionally expressing stronger negative views, whereas current students respond in a more uniformly positive manner.

## 4 Discussion

This study is the first to evaluate student and graduate perceptions of their development of the core attitudes from the British Pharmacological Society's undergraduate pharmacology curriculum. Here, we demonstrate that students and graduates believe that their degrees are enabling their development of these core attitudes, highlighting that undergraduate pharmacology programmes are largely effective in fostering professional behaviors and values, supporting the intended outcomes of the curriculum.

While we observed a higher frequency of negative responses from graduates compared to students, a recent report from PricewaterhouseCoopers listed pharmacology as the fourth highest degree for increasing self‐reported life satisfaction, as well as the third highest for increasing gross earnings after medicine and dentistry and economics [6]. The greater variability and occasional negative responses observed among graduates highlight that some attitudes may be experienced or valued differently once students enter the workplace or progress through their careers. Alternatively, graduates may engage in more critical reflection on their undergraduate degree once faced with real‐world demands.

It is also notable that a disconnect exists between student and graduate self‐perceptions and employer perspectives. While 66.9% (79/118) of students and 67.1% (47/70) of graduates agreed or strongly agreed that they had the “confidence and ability to apply my skills in a real‐world setting”, a survey by the Association of the British Pharmaceutical Industry (ABPI) reported that over half of employer respondents identified the application of scientific, mathematical, and digital knowledge as areas of concern among recruits [7]. This underscores the distinction between perceived and actual preparedness, where confidence does not equate to competence, and highlights the importance of integrating experiential and applied learning opportunities to bridge this gap.

Moreover, the highest proportion of negative responses in both groups was observed for identifying employment opportunities, highlighting a potential gap in career preparation. This is consistent with recent research conducted by ABPI that found young people remain unaware of the full range of roles available within the pharmaceutical industry and the pathways to enter them, potentially limiting their ability to recognize and pursue diverse career options [8].

This challenge may begin even before university, with evidence from preuniversity students showing a perception that a pharmacology degree leads primarily to a single “pharmacologist” career, thereby suggesting that awareness of the breadth of pharmacology‐related careers is limited at entry and may persist beyond graduation [9].

Both students and graduates responded neutrally to core attitude statements “…courage to stand up for my principles under pressure” and “…how to evaluate risk”. Neutral responding is particularly informative in attitudinal research, as it may indicate limited exposure, difficulty recognizing the development of dispositions, or participant uncertainty [10]. Neutral responses provide important curricular signals, highlighting areas where attitude development could be made more explicit to ensure clearer alignment between learning activities and professional behaviors.

A limitation of this study is its reliance on a voluntary online survey, which may be subject to selection bias that affects the representativeness of the sample and the interpretation of responses [11]. Respondents with strong positive or negative views are often more motivated to participate, which may exaggerate the prevalence of more polarized Likert responses and obscure moderate or indifferent attitudes. While online distribution of this survey enabled a broad reach, these methodological challenges underscore that our findings should be interpreted with caution, as the observed distribution of core attitude responses may only reflect the characteristics of those who chose to engage with the survey, rather than a truly representative cross‐section of all pharmacology students and graduates. Moreover, we did not collect information on the specific institutions of respondents to avoid incidental findings about specific institutions. As the survey was disseminated via educators, this study did not track if there was institutional clustering of responses, and it is therefore not possible to determine whether institutions were overrepresented. While this approach supported confidential participation, it limits the ability to assess the institutional distribution and generalisability of responses.

Overall, the predominantly positive responses seen across the attitude statements indicate that educators are effectively supporting the development of pharmacology students and graduates. The areas showing greater variability should not be interpreted as shortcomings, but rather as opportunities for reflective practice in reviewing and refining aspects of their programmes to further strengthen graduate preparedness. The findings of this study can be used as a tool by pharmacology educators to guide curriculum enhancement and refinement and ensure the success of pharmacology graduates.

## Author Contributions

**Sophie G. Reed:** conceptualization, investigation, writing – original draft, methodology, visualization. **Nicola Williams:** conceptualization, investigation, resources. **John Broad:** conceptualization, writing – original draft, visualization, methodology, investigation, project administration. **Aidan Seeley:** conceptualization, formal analysis, visualization, writing – original draft, methodology, investigation, project administration, writing – review and editing.

## Funding

The authors have nothing to report.

## Conflicts of Interest

A. S. and J. B. are members of the Education and Training Committee at the British Pharmacological Society during this study. A. S. was a member of the steering group for the principles for inclusive implementation of the undergraduate pharmacology core curriculum. N.W. is the Professional Development Manager at the British Pharmacological Society.

## Data Availability

The data that support the findings of this study are available from the corresponding author upon reasonable request.
